# Strengthening the Impact of Digital Cognitive Behavioral Interventions Through a Dual Intervention: Proficient Motivational Interviewing–Based Health Coaching Plus In-Application Techniques

**DOI:** 10.2196/34552

**Published:** 2022-05-11

**Authors:** Catherine Serio, Amanda Gabarda, Fatma Uyar-Morency, Valerie Silfee, Justin Ludwig, Eva Szigethy, Susan Butterworth

**Affiliations:** 1 UPMC Health Plan Pittsburgh, PA United States; 2 Happify Health New York, NY United States; 3 Department of Psychiatry University of Pittsburgh Pittsburgh, PA United States

**Keywords:** digital health, mHealth, cognitive behavioral therapy, motivational interviewing, COVID-19, mental health

## Abstract

**Background:**

The COVID-19 pandemic has accelerated the adoption of digital tools to support individuals struggling with their mental health. The use of a digital intervention plus human coaching (“dual” intervention) is gaining momentum in increasing overall engagement in digital cognitive behavioral interventions (dCBIs). However, there is limited insight into the methodologies and coaching models used by those deploying dual interventions. To achieve a deeper understanding, we need to identify and promote effective engagement that leads to clinical outcomes versus simply monitoring engagement metrics. Motivational interviewing (MI) is a collaborative, goal-oriented communication approach that pays particular attention to the language of change and is an effective engagement approach to help people manage mental health issues. However, this approach has been traditionally used for in-person or telephonic interventions, and less is known about the application of MI to digital interventions.

**Objective:**

We sought to provide a dual intervention approach and address multiple factors across two levels of engagement to operationalize a dCBI that combined cognitive behavioral therapy–based techniques and MI-based interactions between the digital health coach (DHC) and user.

**Methods:**

We reviewed hundreds of digital exchanges between DHCs and users to identify and improve training and quality assurance activities for digital interventions.

**Results:**

We tested five hypotheses and found that: (1) users of a dual digital behavioral health intervention had greater engagement levels than users of a noncoached intervention (*P*<.001); (2) DHCs with a demonstrated competency in applying MI to digital messages had more engaged users, as measured by the DHC-to-user message exchange ratio (*P*<.001); (3) the DHC-to-user message exchange ratio was correlated with more engagement in app activities (r=0.28, 95% CI 0.23-0.33); (4) DHCs with demonstrated MI proficiency elicited a greater amount of “change talk” from users than did DHCs without MI proficiency (H=25.12, *P*<.001); and (5) users who were engaged by DHCs with MI proficiency had better clinical outcomes compared to users engaged by DHCs without MI proficiency (*P*=.02).

**Conclusions:**

To our knowledge, this pilot was the first of its kind to test the application of MI to digital coaching protocols, and it demonstrated the value of MI proficiency in digital health coaching for enhanced engagement and health improvement. Further research is needed to establish coaching models in dCBIs that incorporate MI to promote effective engagement and optimize positive behavioral outcomes.

## Introduction

### Background

Over the last several decades, digital technologies have drastically transformed health care delivery and clinical care. From electronic medical records to wearable devices and mobile apps, digital tools have enhanced disease diagnoses and treatment, access to care, and population health management [1,2]. The COVID-19 pandemic has further accelerated digital health adoption, as consumers have increasingly turned to digital health solutions such as telemedicine and digital trackers for medical emergencies or to treat/manage a chronic or mental health condition [3]. While the United States has a long history of mental health concerns, access to treatment has become critical, as the increased psychological strain due to the pandemic has exacerbated this crisis and driven behavioral health referrals to record levels [4,5]. Given the limited availability of mental health providers and the widespread population needs, the possibility of deploying digital behavioral health solutions to wider audiences demands rigorous consideration [6].

Digital cognitive behavioral interventions (dCBIs) have the potential to address unmet behavioral health needs by offering scalable, cost-effective solutions, increasing reach and availability, and allowing consumers to engage and progress at their own pace [7-9]. However, the clinical success of dCBIs is dependent on ample user engagement, and these programs have often faced low levels of adherence and high levels of attrition [10,11]. Researchers in the digital engagement literature have presented nuanced and comprehensive discussions regarding the complexity of different types of engagement, as well as how to view, define, and measure them [10,12-14]. For example, Cole-Lewis et al [13] proposed that engaging with the digital intervention features such as the number of logins, clicks, and time spent (“Little e”), are a critical precursor to engagement in health behaviors such as physical activity or smoking (“Big E”). Yardley et al [12] recognized engagement as multifaceted, presenting two different levels: (1) micro-level engagement—the moment-to-moment engagement with the intervention, including intervention use (eg, number of activities completed) and the user experience (eg, level of user interest and attention when completing activities); and (2) macro-level engagement—the depth of involvement with the behavior change process (eg, extent of motivation for changing behavior) and the link of this engagement to the behavioral goals of the intervention. These definitions help bring clarity to how users interact with a digital solution; however, the engagement metrics presented in the literature fail to include the effects of a key component of many dCBIs: a digital health coach (DHC).

The use of a digital intervention plus human coaching (ie, a “dual” intervention) is gaining momentum in increasing the overall engagement in and clinical effectiveness of dCBIs. In dual programs, the DHC provides support and guidance using asynchronous, chat-based communication. There is substantial evidence in the literature that the addition of health coaching support successfully reinforces adherence to the intervention and improves clinical outcomes for those experiencing mental health issues [7-9,15,16]. However, among those interventions that have specifically measured engagement with a DHC (as opposed to measuring engagement with app features), evaluations are often focused solely on volume, such as the number of messages sent to and from the coach [7,17]. As a result, there is limited insight into methodologies and coaching models used by those deploying dual interventions. To achieve a deeper understanding, we need to establish and promote “effective engagement” versus simply more engagement, with “effective engagement” defined empirically as sufficient engagement with the intervention to achieve the intended outcomes [12].

Motivational interviewing (MI) has been studied for multiple decades and is an effective engagement approach to help people manage mental health issues such as anxiety and depression [18-21]. MI is a collaborative, goal-oriented communication approach that pays particular attention to the language of change [22]. It is the most evidence-based health coaching approach to date with over 1400 clinical trials, standardized and validated tools that measure proficiency and fidelity to the approach, and congruency with the prevailing science of self-determination, self-efficacy, stages of change, and self-perception theories [22-24]. The most compelling research in MI demonstrates that an MI-proficient practitioner can minimize client “sustain talk” (ie, barriers and challenges to change) while evoking “change talk” (ie, desire, ability, reasons, and need for change) [25]. This results in the strengthening of the client’s commitment to change and, in turn, an increase in targeted behaviors and clinical outcomes, such as adopting the practice of CBT exercises and gaining improved mood as a result [25]. However, as this approach has been predominantly used for in-person or telephonic interventions to date, less is known about the application of MI to digital interventions.

### Objective

To date, there is a dearth of literature regarding how or if an MI-based health coaching approach can be successfully integrated into digital coaching and whether it is effective in increasing engagement, eliciting change talk, and improving outcomes in digital settings. Given the evidence supporting MI as an effective approach for engaging people in behavior change, we hypothesized that if a DHC were to incorporate MI into digital messages, they would better engage and activate the user. Therefore, the purpose of this pilot study was to define and articulate an MI-based digital coaching protocol for dCBIs targeting anxiety and depression to better understand the extent to which a dual intervention approach can increase user engagement. A secondary objective was to assess the impact of full proficiency in MI on behavioral health outcomes.

## Methods

### Dual Intervention Model

First, we sought to develop and provide the equivalent of a “dual intervention.” The dual intervention model delivered two concurrent interventions within a mobile app-based dCBI: (1) cognitive behavioral therapy (CBT)–based techniques (eg, relaxation, cognitive reframing, mindfulness); and (2) MI-based interactions between the DHC and the user, including identifying strategies and goals outside the app activities. The mobile app was developed from evidence-based approaches to behavioral health (CBT and mindfulness) by a large health plan in northeast United States that is part of an integrated health care delivery system. The app delivers anxiety or depression programming to users. Individuals choose which program to enroll in, and access to a DHC is dependent upon the client’s health care benefits package or relationship with the health care organization. The coach-enhanced dCBI has been evaluated in diverse care settings including primary care, adolescent care, women’s health, and patient-centered specialty medical homes [26,27].

DHCs received approximately 90 hours of training through an in-house health coach academy. The curriculum was based on national coaching standards and the proprietary health plan Health Coaching Model, which includes a foundation of MI along with other evidence-based health coaching strategies. The model is designed to support coaches in partnering with and empowering their clients to self-manage health behaviors, reach their health and wellness goals, be more productive and resilient, achieve better clinical outcomes, and enhance their overall well-being. After foundational training, the DHCs received an additional training curriculum specific to incorporating MI into the digital coaching platform. A mixed methods training was used with a combination of asynchronous and synchronous training, feedback, mentoring, and assessment using the standardized and validated Motivational Interviewing Competency Assessment (MICA) tool, which was adapted for digital coaching [28]. As DHCs began their interactions with members, they received ongoing skill-building training and quality assurance review assessments, paired with strength-based mentoring and monthly feedback on their digital user interactions.

### Ethics Approval

This quality improvement project was approved by the UPMC Quality Improvement Review Committee (QRC Project ID 2809).

### Testing the Dual Intervention: Five Hypotheses

Building on the model of engagement by Yardley et al [12], we reviewed hundreds of digital exchanges between DHCs and users on an ongoing basis to improve training and quality assurance activities for our mobile app-based dCBI. During this process, we sought to address multiple factors across two levels of engagement to operationalize engagement metrics that incorporated both CBT-based app activities and DHC interactions. Thus, we organized our five hypotheses around micro (ie, engagement within the app) and macro (ie, engagement around behavior change) engagement levels (Table 1) [12].

**Table 1:** Five dual intervention hypotheses.

Engagement			Hypotheses
**Microengagement**			
	Hypothesis 1 (H1)	Users of a dual dCBI^a^ (coaching plus techniques) will have greater engagement than users of a self-guided (techniques only) intervention.	
	Hypothesis 2 (H2)	DHCs^b^ with a demonstrated competency in applying MI^c^ to digital messages will have more engaged users compared to DHCs without MI proficiency, as measured by DHC-to-user message exchange ratio.	
	Hypothesis 3 (H3)	DHC-to-user message exchange ratio (engagement metric) will be correlated with engagement in app activities (number of techniques, days in app in 30 days).	
**Macroengagement**			
	Hypothesis 4 (H4)	DHCs with demonstrated MI proficiency will elicit a greater amount of “change talk” from users than DHCs without MI proficiency.	
	Hypothesis 5 (H5)	Users who were engaged by DHCs with MI proficiency will have better clinical outcomes, indicated by validated mood assessments, as compared to users engaged by DHCs without MI proficiency.	

^a^dCBI: digital cognitive behavioral intervention.

^b^DHC: digital health coach.

^c^MI: motivational interviewing.

### Hypothesis 1

There is evidence to show that coaching support in a digital intervention can positively impact engagement [16,29-31]. We sought to validate this research by analyzing whether there were substantial differences in engagement in app activities between users who participated in a coach-enhanced program and users who participated in the same intervention but self-guided (without a DHC). Self-guided users were selected from a client who opted to not include DHCs as part of their benefits package. To evaluate baseline differences between groups and programs, a chi-square statistic was used to test the difference in the proportion of coached users in groups and programs.

### Hypothesis 2

To test this hypothesis, we randomly selected 50 transcripts with a 30-day exchange period between DHCs and users, intentionally using a cross-section of DHCs across a continuum of experience and training. We then assessed the MI skillset of the DHC using the MICA [25], a standardized, validated tool to assess the competency of clinicians in using the MI approach [25]. For this study, we adapted the MICA scoring methodology by classifying the composite (total) MICA score into quartiles to accommodate the somewhat low DHC-to-user exchanges that occurred in some transcripts (Table 2). Higher MICA scores (and quartiles) indicate a more skilled coach: quartile 4 equates MI proficiency; quartile 3 equals a client-centered level; and quartiles 1 and 2 are considered below client-centered. Two experienced MICA coders double coded 25% of the transcripts to ensure interrater reliability using the quartile method. We also examined the corresponding message ratio between the user and DHC by measuring the ratio of the number of messages from the coach to the number of messages from the user. A lower ratio signifies more interaction from the user (which is preferable).

**Table 2:** Motivational interviewing competency assessment quartiles.

MICA^a^ Score	Quartile	Description
2.0-3.9 (very low)	1	Below client-centered
4.0-5.9 (low)	2	Below client-centered
6.0-7.9 (medium)	3	Client-centered
8.0-10.0 (high)	4	MI^b^ proficient

^a^MICA: Motivational Interviewing Competency Assessment.

^b^MI: motivational interviewing.

### Hypothesis 3

We examined 1128 transcripts during the users’ initial 30-day period of using the app. Inclusion criteria were “coach-engaged users,” defined as users who sent at least one message to their assigned coach. High coach engagement was defined as users who responded with at least one message to every two messages from the DHC (ie, a DHC user ratio of 2.0 or less, N=413). App engagement metrics included the number of techniques and days in app in a 30-day period and were evaluated as dependent measures against coach engagement metrics.

### Hypothesis 4

As previously discussed, change talk is a client utterance during a coaching session that is associated with clinical outcomes in traditional MI-based health coaching interventions [25,32]. A standardized and validated scoring system using the validated Motivational Interviewing Skill Code (MISC) was used to determine a change talk score based on the type of change talk (preparatory or mobilizing) and the strength of the change talk [33].

### Hypothesis 5

This analysis examined differences between clinical outcomes for users who engaged with DHCs with MI proficiency and those who engaged with DHCs without MI proficiency. Anxiety and depression scores were measured within the app via the Generalized Anxiety Disorder 7-item scale (GAD-7) [34] and the Patient Health Questionnaire 8-item scale (PHQ-8) [35]. Clinical success was defined as a reduction of four points [36,37]. Because of this indicator, any user with a baseline score of four or less was not used for this analysis. The groups for DHC proficiency were based on the MICA quartiles, and the first two quartiles were grouped together because the n for quartile 1 was very small, and quartiles 1 and 2 were also similar in terms of ability (ie, less than client-centered). Fisher exact test was used to assess an association between DHC capability and rate of success.

## Results

### Hypothesis 1

During this pilot window, there was a total of 4628 users (Table 3). Out of 3218 users that were enrolled in the anxiety dCBI, 62% (1995/3218) were in the coach-enhanced program. Out of 1410 depression dCBI users, 60% (846/1410) were in the coach-enhanced program (χ^2^=2.4; *P*=.12). Users were between 16 and 87 years of age (mean 40, SD 14) and 70% were female. dBCI app engagement was measured via two metrics: the number of app activities (“techniques”) completed by the user and the total number of days that the user was active in the app. A Kolmogorov-Smirnov statistic was run on coached and self-guided user samples with app engagement metrics as the dependent variable. We found significant group differences between self-guided and coached users in terms of app engagement metrics (*P*<.001). Coached members in both anxiety and depression programs spent more days in the app (*P*<.001) and completed more app activities (*P*<.001).

**Table 3:** Coached versus noncoached engagement.

			Noncoached		Coached		Statistic		*P* value	
**Anxiety**										
	Users, n	1223		1995		N/A^a^		N/A		
	Age (years), mean (SD)	42 (10)		38 (15)		9.068		<.001		
	Gender, n (% female)	773 (64)		1556 (78)		N/A		N/A		
	Techniques, mean (SD)	0.96 (2.77)		5.82 (10.7)		0.43		<.001		
	Days in app, mean (SD)	3.67 (6.37)		11.1(10.7)		0.42		<.001		
**Depression**										
	Users, n	570		840		N/A		N/A		
	Age (years), mean (SD)	43 (10)		39 (15)		6.007		<.001		
	Gender, n (% female)	342 (60)		605 (72)		N/A		N/A		
	Techniques, mean (SD)	1.23 (2.64)		5.3 (9.1)		0.31		<.001		
	Days in app, mean (SD)	4.07 (6.9)		10.2 (10.6)		0.34		<.001		
**Total**										
	Users, n	1793		2835		N/A		N/A		
	Age (years), mean (SD)	42 (10)		39 (15)		8.161		<.001		
	Gender, n (% female)	1115 (62)		2161 (76)		N/A		N/A		
	Techniques, mean (SD)	1.05 (2.7)		5.67(10.3)		0.39		<.001		
	Days in app, mean (SD)	3.8 (6.5)		10.8 (10.7)		0.40		<.001		

^a^N/A: not applicable.

### Hypothesis 2

A Pearson product-moment correlation examined the relationship between the MICA quartile and message ratio and found that higher MICA quartiles had a lower message ratio with their users (*r*=–0.79, 95% CI–0.87 to –0.66). A Kruskal-Wallis nonparametric statistic was also calculated to detect differences in message ratios based on the MICA quartile group (Table 4). The *P* value for the Kruskal-Wallis test was significant (*P*<.001); the mean message ratio improved with increasing MI proficiency, indicating that coaches who were more skilled experienced more interaction/engagement with their users.

**Table 4:** Coach quartile and average message ratio.

		Message transcripts (N=50)	Average message ratio^a^	Standard deviation	Median	Correlation ratio		*P* value (Kruskal-Wallis test group differences)	
**MICA^b^** **score quartile**							0.79		*P*<.001
	1	10	3.83	0.99	3.58				
	2	14	2.55	0.70	2.68				
	3	12	1.95	0.31	1.94				
	4	14	1.44	0.36	1.33				

^a^Average message ratio is better when exchange number is lower.

^b^MICA: Motivational Interviewing Competency Assessment.

### Hypothesis 3

A Pearson product-moment correlation was used to examine differences in app engagement between users who had high engagement with their assigned DHC and those who had low engagement with their DHC (Table 5). Users who were more engaged with their coach completed a greater number of techniques (*r*=0.28, 95% CI 0.23-0.33) and spent more days in the app (*r*=0.37, 95% CI 0.32-0.42). A Kruskall-Wallis nonparametric test evaluated group differences in app engagement metrics within high and low coach engagement and found that both techniques and days in app were significant (*P*<.001). Overall, app engagement increased with greater coach engagement, indicating greater rates of response to coach messages.

**Table 5:** Coach engagement and app engagement.

Coach engagement			Participants (N=1128)		Average number of techniques		Standard deviation		Median		Correlation ratio			Kruskal-Wallis test (group differences)			*P* value	
**Number of techniques**												0.28			92.4			<.001
	Low^a^	715		7.46		9.4		5										
	High^b^	413		15.56		18.3		9										
**Days in the app**												0.37			174.9			<.001
	Low^a^	715		15.43		9.73		15										
	High^b^	413		23.15		8.71		28										

^a^Low engagement=digital health coach (DHC):user message ratio>2.0

^b^High engagement=DHC:user message ratio<2.0

### Hypothesis 4

The average change talk score increased as the MI proficiency increased for the DHCs. A Kruskal-Wallis nonparametric statistic was calculated to determine whether there were substantial differences in change talk in user responses between users who interacted with an MI-proficient DHC and those who interacted with DHCs below MI proficiency (Table 6). The test indicated that there was a difference in change talk scores with higher MI proficiency (H=25.12, *df*=2; *P*<.001).

**Table 6:** Change talk score by the Motivational Interviewing Competency Assessment (MICA) score^a^.

MICA score	Transcripts (N=30)	Min	25th percentile	Median	75th percentile	Max	Mean	Standard deviation
Low	10	3	5	7.5	8	12	7.2	2.86
Medium	10	11	14	17	18	20	15.8	3.16
High	10	25	27	35.5	45	51	36.6	9.57

^a^Kruskal-Wallis (group differences): *P*<.001.

### Hypothesis 5

Users who were engaged by DHCs with MI proficiency had a higher success rate (Table 7; *P*=.02). This result was also significant for users in the anxiety program (*P*=.03), while the results for users in the depression program trended toward significance (*P*=.06); this cohort had a much lower volume, particularly those users interacting with a highly skilled DHC. A Cochran-Mantel-Haenszel (CMH) trend test was also used to determine whether there was a linear trend between DHC proficiency and success. The CMH trend test showed similar results for the overall and anxiety discussion groups (*P*=.02 and *P*=.007, respectively). These findings indicate that there was an increased rate of clinical success among users who engaged with more skilled DHCs.

**Table 7:** Program success based on MICA quartile.

Reduction		Yes, n (%)	No, n (%)	Overall independence *P* value (CMH^a^ test)		Trend *P* value (CMH test)	
**Depression and anxiety**					.02		.02
	MICA^b^ quartiles 1 and 2	36 (46)	43 (54)				
	MICA quartile 3	37 (62)	23 (38)				
	MICA quartile 4	21 (75)	7 (25)				
**Anxiety**					.03		.006
	MICA quartiles 1 and 2	23 (41)	33 (59)				
	MICA quartile 3	20 (56)	16 (44)				
	MICA quartile 4	19 (73)	7 (27)				
**Depression**					.06		.16
	MICA quartiles 1 and 2	13 (57)	10 (43)				
	MICA quartile 3	17 (71)	7 (29)				
	MICA quartile 4	2 (100)	0 (0)				

^a^CMH: Cochran-Mantel-Haenszel

^b^MICA: Motivational Interviewing Competency Assessment.

## Discussion

### Principal Findings

The emergence of digital health interventions has afforded the opportunity to address behavioral health crises in the United States by improving access to care and reaching more people who are struggling with mental health issues. Evidence continues to demonstrate that dCBIs can effectively help people manage anxiety and depression, and these effects increase with greater intervention engagement [9,15,30,38]. While DHCs are emerging as important mediators to this, it remains critical to better understand how to strengthen the impact of DHCs by focusing on the quality of engagement rather than simply on quantity (ie, volume of messages sent). This pilot study tested the hypothesis that systematically applying an evidence-based coaching approach (MI) to digital coaching protocols would enhance dCBI engagement and outcomes.

First, our results underscored the importance of a dual intervention approach, as our findings were consistent with literature demonstrating that combining technology with coaching elicits greater app engagement compared to self-guided interventions [30,31]. Moreover, we validated that a standardized MI coaching protocol can be effectively integrated into a digital intervention. MI is considered a best practice communication approach with extensive evidence supporting its ability to positively impact behavioral outcomes [18-20]. Given the historical use of MI for in-person or telephonic interventions, it is encouraging to see that not only can it be applied to an asynchronous chat but also that increased user engagement with a DHC was correlated with spending more days in the app and completing more app techniques. This is critical, as evidence suggests a dose-effect relationship between intervention engagement and health improvement [13]. Therefore, the more a user practices app techniques and interacts with their DHC, the more CBT they receive, and the better they ultimately feel. We found these effects were even greater when a user interacted with a DHC who was fully proficient in MI.

An additional question we aimed to answer was what level of MI proficiency a digital coach needs to be effective. An MI-proficient DHC empowers and activates the user while a client-centered DHC engages and responds to user needs in a supportive way. DHCs in this pilot who empowered and activated users had more interactions and elicited a greater amount of “change talk,” which allowed the DHC to strengthen motivation and the desire to change by evoking, reflecting, affirming, summarizing, or elaborating on the change talk. This in turn allowed for more dialogue in the direction of change and empowered the user by increasing language that indicates a sense of self-efficacy and personal agency. As a result, users with more highly skilled coaches completed more app activities, spent more days in the app, and had better clinical outcomes. While these benefits were demonstrated among client-centered DHCs, we found that MI-proficient DHCs produced the greatest outcomes.

### Implications for Practice

The success of dCBIs is dependent on robust user engagement. Unfortunately, these programs often demonstrate low levels of engagement and high dropout rates [31,39]. Results from this pilot suggest that developing an MI-proficient DHC skill set is critical to enhancing dCBI engagement, health behavior change, and behavioral health outcomes. Reaching MI proficiency depends not only on initial comprehensive training but also on ongoing competency assessment, feedback, mentoring, and skill building [40]. Unfortunately, while organizations are investing in bringing digital tools into their clinical workflows, they are not investing the time or resources to reach this level of proficiency among those delivering the intervention. While employing DHCs who are client-centered may help to move the needle, MI proficiency may be necessary to optimize the return on the digital health investment.

### Conclusion

DHCs have emerged as a new care role that can extend our reach and ability to support individuals struggling with their mental health. To our knowledge, this pilot was the first of its kind to test the application of MI to digital coaching protocols, demonstrating the value of MI proficiency in digital health coaching for enhanced engagement and health improvement. To have a significant impact on the field of dCBIs, organizations investing in digital health have key decisions to make regarding DHC training and support, as clinical behavioral health outcomes may depend on making a stronger commitment to strengthen the exchange between users and DHCs.

## Abbreviations

CBT: cognitive behavioral therapy
CMH: Cochran-Mantel-Haenszel
dCBI: digital cognitive behavioral intervention
DHC: digital health coach
GAD-7: Generalized Anxiety Disorder 7-item scale
MI: motivational interviewing
MICA: Motivational Interviewing Competency Assessment
MISC: Motivational Interviewing Skill Code
PHQ-8: Patient Health Questionnaire 8-item scale
